# Comparing cranial-caudal-medial and medial–lateral approaches for laparoscopic right hemicolectomy: a propensity score-matched analysis

**DOI:** 10.1186/s12957-024-03465-8

**Published:** 2024-07-22

**Authors:** Jie Wang, Jiajie Zhou, Yifan Cheng, Shuai Zhao, Ruiqi Li, Chenkai Zhang, Yayan Fu, Longhe Sun, Jun Ren, Daorong Wang

**Affiliations:** 1 Northern Jiangsu People’s Hospital Affiliated to Yangzhou University, Yangzhou University, Yangzhou, Jiangsu, 225001 China; 2 Northern Jiangsu People’s Hospital, Clinical Teaching Hospital of Medical School, Nanjing University, Yangzhou, 225001 China; 3 The Forth People’s Hospital of Taizhou, Taizhou, 225300 China; 4 Northern Jiangsu People’s Hospital, Yangzhou, 225001 China; 5 Yangzhou Key Laboratory of Basic and Clinical Transformation of Digestive and Metabolic Diseases, Yangzhou, Jiangsu, 225001 China; 6 General Surgery Institute of Yangzhou, Yangzhou University, Yangzhou, Jiangsu, 225001 China

**Keywords:** Cranial-caudal-medial, Medial–lateral, Right-sided colon cancer, Laparoscope, Complete mesocolic excision

## Abstract

**Background:**

The cranial-caudal-medial approach (CCMA) has been proposed for laparoscopic right hemicolectomy nowadays. This study aimed to investigate the safety and oncological efficacy of CCMA in the treatment of right-sided colon cancer compared to the medial–lateral approach (MLA).

**Methods:**

Patients diagnosed with right-sided colon cancer were included from February 2015 to June 2018, retrospectively, dividing into the CCMA group and the MLA group. We compared the basic characteristics and the short-term and long-term outcomes in two groups.

**Results:**

Two hundred and ninety-six patients were included in this study. The baseline characteristics were similar in two groups. Compared with MLA group, CCMA group exhibited shorter operation time (136.3 ± 25.3 min vs. 151.6 ± 21.5 min, *P* < 0.001), lower estimated blood loss (44.1 ± 15.2 ml vs. 51.4 ± 26.9 min, *P* = 0.010), and more harvested lymph nodes (18.5 ± 7.1 vs. 16.5 ± 5.7, *P* = 0.021). The 5-year overall survival (OS) rate for the CCMA group was 76.5%, and the 5-year disease-free survival (DFS) rate was 72.3%, both of which were not inferior to the MLA group. No significant difference was found between two groups in terms of other clinical parameters.

**Conclusion:**

The CCMA in laparoscopic right hemicolectomy is safe and feasible, making the anatomical plane clearer. This approach can shorten the operation time, reduce intraoperative blood loss, harvest more lymph nodes, and yield satisfactory oncological outcomes.

**Supplementary Information:**

The online version contains supplementary material available at 10.1186/s12957-024-03465-8.

## Introduction

Colorectal cancer ranks as the third most prevalent cancer worldwide and the second leading cause of mortality [1]. In 2009, Professor Hohenberger introduced complete mesocolic excision (CME) [2], which became the established surgical method for right-sided colon cancer (RSC) [3, 4]. Laparoscopic right hemicolectomy (LRH) is a complex and challenging surgery, characterized by a considerably steeper learning curve, primarily due to the anatomical variations in blood vessels and the complexity of surrounding tissue structures [5]. LRH has undergone a series of modifications and developments, taking into account surgical safety and oncological outcomes.

Presently, several approaches were performed in LRH, such as the cranial approach, caudal approach, medial–lateral approach (MLA), and a combination of these approaches [6, 7]. The MLA is the classic method for LRH. In this approach, the tumor-feeding blood vessels are first ligated. Then, the intestinal segment is mobilized where the tumor is situated. Therefore, the MLA complies with the “no-touch” principle [8]. Nevertheless, this approach requires advanced surgical skills. Beginners may require a longer time to become proficient [9, 10]. To simplify the surgical procedures of LRH, several mixed approaches have been proposed. The cranial-to-caudal approach was proposed by Matsuda et al. in 2015 to facilitate the proper and easy management of the gastrocolic trunk [11]. In 2016, Li et al. investigated the application of the caudal-to-cranial approach [12]. Due to the recent advancements in CME, minimally invasive techniques, and membrane anatomy techniques, the application of the mixed approach in LRH has garnered increasing attention [13–16].

Besides the aforementioned approaches, Yao et al. reported the cranial-caudal-medial approach (CCMA) to accomplish CME in a counterclockwise direction [17]. Although various approaches for LRH were reported in the literature, few studies focus on CCMA. Moreover, most studies lack long-term outcomes. Since 2015, the CCMA and MLA are two common approaches used by our surgical team for LRH. Therefore, this retrospective study examined the short-term results and long-term survival outcomes of the CCMA and MLA.

## Materials and methods

### Patients

Four hundred and seventy patients diagnosed with RSC were included consecutively in our study at Northern Jiangsu People's Hospital from February 2015 to June 2018. The inclusion criteria were specified as follows: (1) isolated malignant tumors located in the ileocecal, ascending colon, or hepatic flexure, (2) diagnosis confirmed by electron colonoscopy and pathology, (3) laparoscopic surgery, and (4) CCMA or MLA performed. The exclusion criteria were specified as follows: (1) open surgery, (2) emergency surgery, (3) history of cancer, and (4) multiple primary tumors or distant metastases.

Every surgical operation was carried out by the same team of surgeons. After fully understanding advantages and disadvantages of CCMA and MLA, patients freely choose the surgical approach. All participants have signed informed consent forms. This study was approved from Ethics Committee of Northern Jiangsu People's Hospital (No. 2016KY-022).

### Data collection

We collected basic characteristics, perioperative data, and follow-up data. Basic characteristics included sex, age, body mass index (BMI), prior abdominal surgery, tumor location, American Society of Anesthesiologists (ASA) classification, tumor size, American Joint Committee on Cancer (AJCC) stage and neoadjuvant therapy. The AJCC stage represented the pathological stage. Perioperative data was obtained from surgical records and pathological reports, including operation time, estimated blood loss, conversion to open surgery, anastomosis method, time to first gas passing, time to first stool passing, time to first fluid diet, postoperative hospitalization and pathology data. Follow-up data was collected during clinic visits and telephone follow-up, which comprised 5-year overall survival (OS) rate and 5-year disease-free survival (DFS) rate. Overall postoperative complications included short-term postoperative complications, including complications ranging Grade I-II to III-IV as per the Clavien-Dindo classification system, within the initial 30 days post-surgery or throughout the complete hospitalization period if it exceeded 30 days. The primary outcomes were OS and DFS. secondary outcomes were overall postoperative complications.

### Surgical approaches

**Fig. 1:**
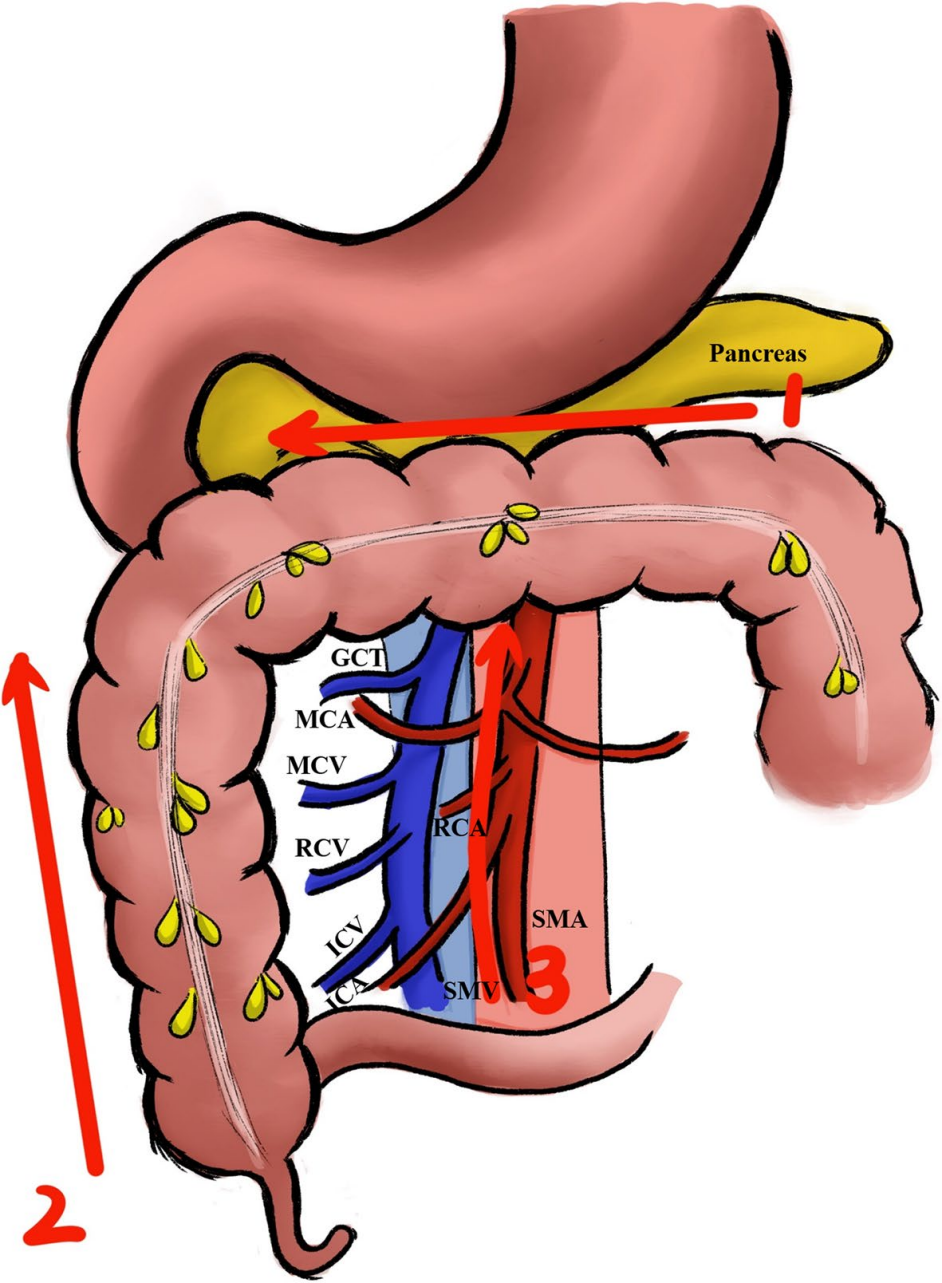
The cranial-caudal-medial approach. Abbreviations: GCT gastrocolic trunk; MCA middle colon artery; MCV middle colon vein; SMV superior mesenteric vein; right colic artery RCA; right colic vein RCV; ICV ileocolic vein; ICA ileocolic artery; SMA superior mesenteric artery

The surgical procedure for the CCMA group aligned with the description in the study by Yao et al. [17]. Step 1: The gastrocolic ligament was split and the anterior leaf of the transverse mesocolon was dissected in the sub-pyloric area. Then, the surgeon proceeded to dissect along the gastric omental vessels, entering the fusion space between the mesogastrium and the transverse mesocolon. After exposing the right gastroepiploic vein (RGEV), the surgeon proceeded to dissect along this vein to separate the branches of the gastrocolonic trunk, and to expose the middle colonic vein (MCV). Step 2: Surgeon exposed the root of the ileocecal mesentery. Subsequently, the surgeon explored the retroperitoneal plane, which consisted of Toldt’s space and prerenal space, continuing until reaching the surface of the duodenum and pancreas. Step 3: The ileocolic vessel was identified, and the lymph nodes (LNs) were dissected along the superior mesenteric vein (SMV) or artery (SMA). Subsequently, the surgeon ligated the ileocolic vessels, right colic vessels, and middle colic vessels (or the right branch of the middle colic vessels) at their roots. Finally, the right-sided colon was divided with an endo-GIA. The surgeon removed the specimen and reconstructed the gastrointestinal tract (Fig. 1).

#### MLA

The surgical procedure for the MLA group complied with Feng et al.’s study [18]. The initial step involved the identification of the ileocolic vessel. The dissection proceeded along the SMV or SMA. The surgeon ligated the bases of the central vessels and exposed Toldt’s space and prerenal space. Subsequently, a complete dissection of the colonic hepatic flexure was made along the fusion area. Lastly, the hepatic flexure and lateral attachments of the ascending colon were mobilized. The surgeon divided the right colon with an endo-GIA and reconstructed the gastrointestinal tract.

### Statistical analysis

SPSS 26.0 (SPSS, Chicago, Illinois, USA) were utilized for data analysis. Categorical data were expressed in percentages (%). Accuracy in comparative analysis was assessed using Fisher's exact test or the χ2 test. Continuous variables following normal distribution were evaluated with the student’s *t*-test and reported as mean ± standard deviation (mean ± SD). Non-normally distributed continuous variables were examined using nonparametric tests and were shown as median (interquartile range). Survival outcomes analyzed with the Kaplan–Meier method, and hazard ratios (HR) with 95% confidence intervals (CI) were computed via Cox regression. A P-value of < 0.05 was deemed significant. For the propensity score model, variables such as age, sex, BMI, ASA classification, tumor location, and prior abdominal surgery were used in a 1:1 nearest neighbor matching algorithm, with a caliper set at 0.2 standard deviations for propensity score-matched (PSM) analysis.

## Results

### Basic characteristics of the patients

The study initially screened 470 patients with RSC, but excluded 174 cases, ultimately including 296 cases for analysis. After PSM, 238 patients (CCMA, n = 119; MLA, *n* = 119) were finally analyzed (Supplementary Fig. 1). No significant differences in baseline characteristics were observed between the two groups following PSM (Table 1).

**Table 1:** Basic characteristics

Characteristics	Before PSM			After PSM		
	**CCMA (*n* = 135)**	**MLA (*n* = 161)**	***P***	**CCMA (*n* = 119)**	**MLA (*n* = 119)**	***P***
**Age (years), mean (SD)**	62.6 (8.9)	65.2 (8.9)	0.013*	62.1 (8.5)	62.2 (9.0)	0.912
**Sex (n, %)**		93 (57.8) 68 (42.2) 22.5 (2.8) 23 (14.3) 56 (34.8) 76 (47.2) 29 (18.0) 44 (27.3) 62 (38.5) 55 (34.2)	0.558		68 (57.1) 51 (42.9) 22.8 (2.9) 13 (10.9) 45 (37.8) 56 (47.1) 18 (15.1) 30 (25.2) 48 (40.3) 41 (34.5)	0.896
**Male**	73 (54.1)			66 (55.5)		
**Female**	62 (45.9)			53 (44.5)		
**BMI (kg/m2), mean (SD)**	22.7 (3.4)		0.593	22.8 (3.5)		0.899
**Prior abdominal surgery (n, %)**	17 (12.6)		0.734	15 (12.6)		0.841
**ASA classification (n, %)**			0.378			0.894
**I**	56 (41.5)			48 (40.3)		
**II**	61 (45.2)			55 (46.2)		
**III**	18 (13.3)			16 (13.5)		
**Tumor location, n (%)**			0.509			0.790
**Ileocecal junction**	29 (21.5)			26 (21.8)		
**Ascending colon**	56 (41.5)			48 (40.3)		
**Hepatic flexure**	50 (37.0)			45 (37.9)		
**Tumor size (n, %)**			0.473			0.784
** ≥ 5 cm**	49 (36.3)	66 (41.0)		39 (32.8)	42 (35.3)	
** < 5 cm**	86 (63.7)	95 (59.0)		80 (67.2)	77 (64.7)	
**AJCC stage (n, %)**			0.671			0.866
**I**	26 (19.3)	30 (18.6)		19 (16.0)	21 (17.6)	
**II**	57 (42.2)	61 (37.9)		59 (49.6)	55 (46.2)	
**III**	52 (38.5)	70 (43.5)		41 (34.4)	43 (36.2)	
**Neoadjuvant therapy (n, %)**	35 (25.9)	48 (29.8)	0.517	29 (24.4)	32 (26.9)	0.767

Results are expressed as n (%) or mean ± SD

*Abbreviations: PSM* propensity score-matched, *CCMA* cranial-caudal-medial approach, *MLA* medial–lateral approach, *BMI* body mass index, *ASA* American Society of Anesthesiologists, *AJCC* American Joint Committee on Cancer

^*^P < 0.05

### Perioperative outcomes

In the CCMA group, operation time was 136.3 ± 25.3 min, significantly less (*P* < 0.001) compared to the MLA group, which was 151.6 ± 21.5 min. The estimated blood loss for the CCMA group was 44.1 ± 15.2 mL, significantly less (*P* < 0.05) compared to the MLA group, which was 51.4 ± 26.9 mL. Furthermore, the number of harvested LNs in the CCMA group was 18.5 ± 7.1, significantly higher (*P* < 0.05) compared to the MLA group, which was 16.5 ± 5.7. In addition, two patients in the CCMA group were converted to open surgery due to adhesions. Whereas there were four cases in the MLA group, with two due to bleeding and two due to adhesions. No significant differences were observed in other perioperative clinical parameters and pathologic data between the two groups (*P* > 0.05) (Table 2).

**Table 2:** Intraoperative and postoperative outcomes

Characteristics	CCMA group (*n* = 119)	MLA group (*n* = 119)	*P*
Operation time (min), mean (SD)	136.3 (25.3)	151.6 (21.5)	< 0.001**
Estimated blood loss (ml), mean (SD)	44.1 (15.2)	51.4 (26.9)	0.010*
Conversion to open surgery (n, %)	2 (1.7%)	4 (3.4%)	0.683
Anastomosis method (n, %)			0.584
Intracorporeal Anastomosis	76 (63.9)	81 (68.1)	
Extracorporeal Anastomosis	43 (36.1)	38 (31.9)	
Time to first gas passing (d), mean (SD)	2.6 (1.2)	2.7 (1.0)	0.555
Time to first stool passing (d), mean (SD)	4.4 (1.2)	4.5 (1.3)	0.372
Time to first fluid diet (d), mean (SD)	3.8 (1.3)	3.9 (1.3)	0.267
Postoperative hospitalization (d), mean (SD)	10.1 (2.3)	10.5 (2.6)	0.227
Number of harvested LNs, mean (SD)	18.5 (7.1)	16.5 (5.7)	0.021*
Number of positive LNs, median (IQR)	1 (0, 3)	1 (0, 3)	0.269
Perineural invasion (n, %)	23 (19.3%)	24 (20.2%)	0.871
Vascular invasion (n, %)	17 (14.3%)	22 (18.5%)	0.381
Tumor differentiation (n, %)			0.303
Well	13 (10.9%)	6 (5.0%)	
Moderate	53 (44.5%)	52 (43.7%)	
Poor	15 (12.6%)	21 (17.7%)	
Mucous	38 (31.9%)	40 (33.6%)	
Pathological T stage (n, %)			0.662
T1	7 (5.9%)	10 (8.4%)	
T2	12 (10.1%)	14 (11.8%)	
T3	62 (52.1%)	53 (44.5%)	
T4	38 (31.9%)	42 (35.3%)	
Pathological N stage (n, %)			0.912
N0	65 (54.6%)	68 (57.1%)	
N1	40 (33.6%)	37 (31.1)	
N2	14 (11.8%)	14 (11.8%)	
Adjuvant therapy	66 (55.5)	62 (52.1)	0.697

*Abbreviations: CCMA* cranial-caudal-medial approach, *MLA* medial–lateral approach, *LNs* lymph nodes

Results are expressed as n (%) or mean ± SD or median (IQR)

*P < 0.05

**P < 0.001

### Complications

The rate of overall complications was 15.1% and 13.4% in the CCMA and MLA groups, respectively. No significant differences in complications were observed between the two groups (Table 3). Serious complications, including anastomotic leakage and postoperative bleeding, occurred in two cases in the CCMA group and three cases in the MLA group. The 30-day mortality rate was zero in both groups.

**Table 3:** Overall postoperative complications

Characteristics	CCMA group (*n* = 119)	MLA group (*n* = 119)	*P*
Overall postoperative complications (n, %)	18 (15.1%)	16 (13.4%)	0.853
I–II (Clavien–Dindo) (n, %)	16 (13.4%)	14 (11.8%)	0.845
Wound infection (n, %)	3 (2.5%)	1 (0.8%)	
Pneumonia (n, %)	1 (0.8%)	4 (3.4%)	
Urinary retention (n, %)	5 (4.2%)	2 (1.7%)	
Ileus (n, %)	5 (4.2%)	4 (3.4%)	
Chylous fistula (n, %)	5 (4.2%)	6 (5.0%)	
III–IV (Clavien–Dindo) (n, %)	2 (1.7%)	3 (2.5%)	1.000
Anastomotic leakage (n, %)	1 (0.8%)	1 (0.8%)	
Postoperative bleeding (n, %)	1 (0.8%)	2 (1.7%)	

*Abbreviations: CCMA* cranial-caudal-medial approach, *MLA* medial–lateral approach

Results are expressed as n (%)

### Survival analysis

**Fig. 2:**
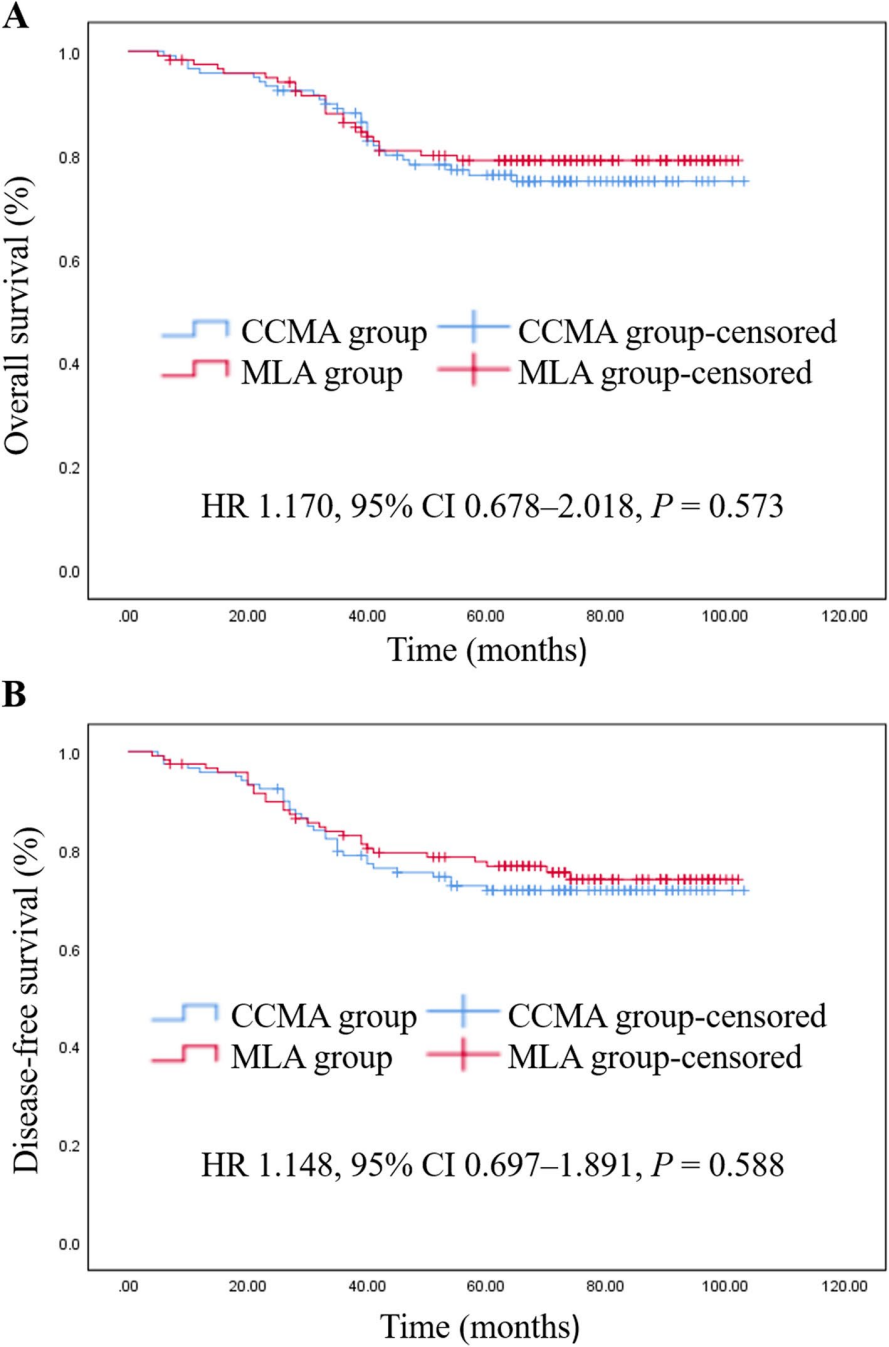
Kaplan–Meier survival curve. (**A**) The 5-year overall survival rate. (**B**) The 5-year disease-free survival rate. Abbreviations: CCMA cranial-caudal-medial approach; MLA medial–lateral approach; HR hazard ratio; CI confidence interval

The median follow-up period was 67 months with an IQR of 42.00–81.00 months. Among all the participants, 52 patients died; among them, 28 patients were included in the CCMA group (28/119, 23.5%) and 24 patients in the MLA group (24/119, 20.2%). The 5-year OS rates were 76.5% for the CCMA group and 79.8% for the MLA group (HR 1.170, 95% CI 0.678–2.018, *P* = 0.573) (Fig. 2A). The 5-year DFS rates were 72.3% for the CCMA group and 75.6% for the MLA group (HR 1.148, 95% CI 0.697–1.891, *P* = 0.588) (Fig. 2B). Supplementary Table 2 shows the recurrence patterns in the two groups.

### Predictors of OS and DFS

Independent predictors were identified by Univariate and multivariate Cox regression. The independent predictors of OS were age (HR 1.043; 95% CI: 1.005–1.083; *P* = 0.028), nerve invasion (HR 2.140; 95% CI: 1.119–4.091; *P* = 0.021), vascular invasion (HR 2.948; 95% CI: 1.522–5.711; *P* = 0.001), pathological TNM stage III (HR 2.298; 95% CI: 1.155–4.572; *P* = 0.018). The independent predictors of DFS were vascular invasion (HR 2.301; 95% CI: 1.062–4.984; *P* = 0.035), pathological TNM stage III (HR 3.425; 95% CI: 1.398–8.390; *P* = 0.007). Surgical approach did not independently correlate with survival outcomes (Supplementary Table 1).

## Discussion

Our study investigated the oncological outcomes of CCMA in LRH, which has been less reported in the previous literature. 470 patients were included in a comprehensive analysis and examined the feasibility, safety, and oncological outcomes of CCMA. After PSM, our results showed that CCMA not only reduced operation time and estimated blood loss, but also harvested more LNs compared with MLA. CCMA also yielded a satisfactory long-term survival.

Surgical approaches for LRH have been widely studied by colorectal surgeons and multiple approaches have been proposed with the development of laparoscopic and CME techniques [19–21]. CCMA is one of them, and this approach was developed to address the limitations of a single approach by surgeons combining the advantages of multiple approaches. Our team has become proficient in the CCMA and perform this technology routinely in LRH. Clinical practice has shown that CCMA have unique advantages in reducing the difficulty and risk of surgery, thanks to clearer anatomical levels. However, unlike MLA, CCMA contradicts the principle of "no-touch", which is the main controversy of this approach [22]. Therefore, we performed a retrospective analysis to investigate the clinical outcomes of CCMA compared with MLA.

In this study, both the operation time and estimated blood loss were significantly reduced in CCMA. This finding aligned with the results reported by Yang Y et al. and reflected, to some extent, the convenience and surgical safety of this technique [23]. The surgical challenge in LRH is the correct surgical plane and precise vascular dissection. In CCMA, the surgeon first mobilizes the colon along clear anatomical landmarks so that the anterior space of the pancreatic duodenum can be easily accessed. The right gastric omental vessels and accessory right colon veins, which are anatomic landmarks for gastrocolonic trunk, are then exposed. Finally, vascular ligation and lymph node dissection are accomplished. By choosing reasonable gaps, CCMA facilitates the clarification of major vascular structures, increases the smoothness of surgical procedure, and reduces intraoperative injuries [17]. The first step of CCMA is similar to the surgical procedure of distal gastric cancer radical surgery. Standardizing the handling of the subpyloric area enables rapid opening of the gastrocolic omentum, exposure of the hepatic flexure, precise tumor localization, which is then followed by the second step to accurate identify of the Toldt’s space. By completely separating the right hemicolon through steps one and two, surgeons are provided with a greater operating space to optimize the vascular skeletonization process based on various anatomical landmarks. The combination of these advantages collectively contributes to the shortening of the operation time.

Increased number of harvested LNs is another advantage of CCMA found in our study. Lykke et al. evaluated the data of 13,766 patients with colon cancer and reported that a lymph node yield of more than 12 indicates an improvement in survival rate [24]. The advantages of CCMA in lymph node dissection are mainly due to the following two factors. Firstly, in CCMA, surgeons could easily enter the Toldt’s space. In this vascular-free plane, complete lymph node dissection was performed and the effect of intraoperative blood loss on the surgical field of vision was decreased. Secondly, in patients with ileocolic artery passing through SMV from the dorsal side, MLA could not clean LNs from the mesenteric root, whereas CCMA could achieve maximum dissection range. Furthermore, this approach has advantages in handling the gastrocolic trunk and its branches. The findings of this study mostly align with those of previous researches [22, 25].

The short-term efficacy is one of the main observational indicators. In this study, there were no significant differences between the two groups in terms of time to first gas passing, time to first stool passing, time to first fluid diet, and postoperative hospitalization. Previous study has reported that the rate of overall postoperative complications in right hemicolectomy with CME was 13.5% approximately [26]. Ileus and chylous fistula are common complications of LRH. In our study, no significant differences were observed in overall postoperative complications (15.1% VS 13.4%) between the two groups.

Oncologic outcomes of CCMA have been a concern for some time. Some scholars have proposed that flipping or compressing the malignancy prior to ligating the vascular ligament has the potential to promote tumor cell proliferation. Nonetheless, the advantages of the “no-touch” principle have not been fully demonstrated, and the potential oncologic benefits of the MLA remain hypothetical. A recent large-scale randomized controlled trial (JCOG1006) reported that techniques following the principle of "no-touch" in open surgery were not superior to conventional techniques [27]. In our study, all patients underwent laparoscopic surgery and we counted the long-term survival of both groups. Notably, the difference in 5-year-OS rates (76.5% vs. 79.8%) and DFS rates (72.3% vs. 75.6%) between the CCMA and MLA groups were not statistically significant. Univariate and multivariate Cox regression analyses also indicated that surgical approach did not independently correlate with survival. Our findings suggest that the use of CCMA in LRH with CME can yield equally satisfactory oncologic outcomes compared with MLA.

Presently, our study has some limitations that should be addressed. This is a single-centered and retrospective study. Therefore, the number of patients was relatively small. Multicentered and large-scale clinical studies are warranted in the future to verify our results. The strength of this study lies in the PSM analysis, which balances all confounding factors between the two compared groups, minimizing significant biases typically present in conventional analyses. In contrast to prior studies, our study provides long-term follow-up data and a certain basis for the long-term survival of patients undergoing LRH via the CCMA. Based on the abovementioned results, CCMA can be considered safe and effective for LRH. The short- and long-term clinical effects of CCMA are good and warrant further clinical promotion and application.

## Conclusion

The CCMA theory adheres to the CME concept. CCMA can address the limitations due to the intraoperative vascular anatomy and decrease the extent of intraoperative damage. This surgical approach exhibits unique advantages and good reproducibility and is propagable for treatment of patients with RSC.

### Supplementary Information

Supplementary Material 1.

## Data Availability

The data used to support the findings of this study are available from the corresponding author upon request.
