# Supplementary material for: Comparing cranial-caudal-medial and medial–lateral approaches for laparoscopic right hemicolectomy: a propensity score-matched analysis
Source: World J Surg Oncol. 2024 Jul 22;22:187. doi: 10.1186/s12957-024-03465-8 (PMC11265123; doi:10.1186/s12957-024-03465-8)
Supplement: Supplementary file 1 — Supplementary Material 1. [file 12957_2024_3465_MOESM1_ESM.docx]

**Supplementary Data**

**Figure legends**

**Supplementary Figure 1. Clinical data selection flowchart.**

Abbreviations: CCMA cranial-caudal-medial approach; MLA medial-lateral approach.

**Supplementary Figure 1. Clinical data selection flowchart (Supplementary data).**


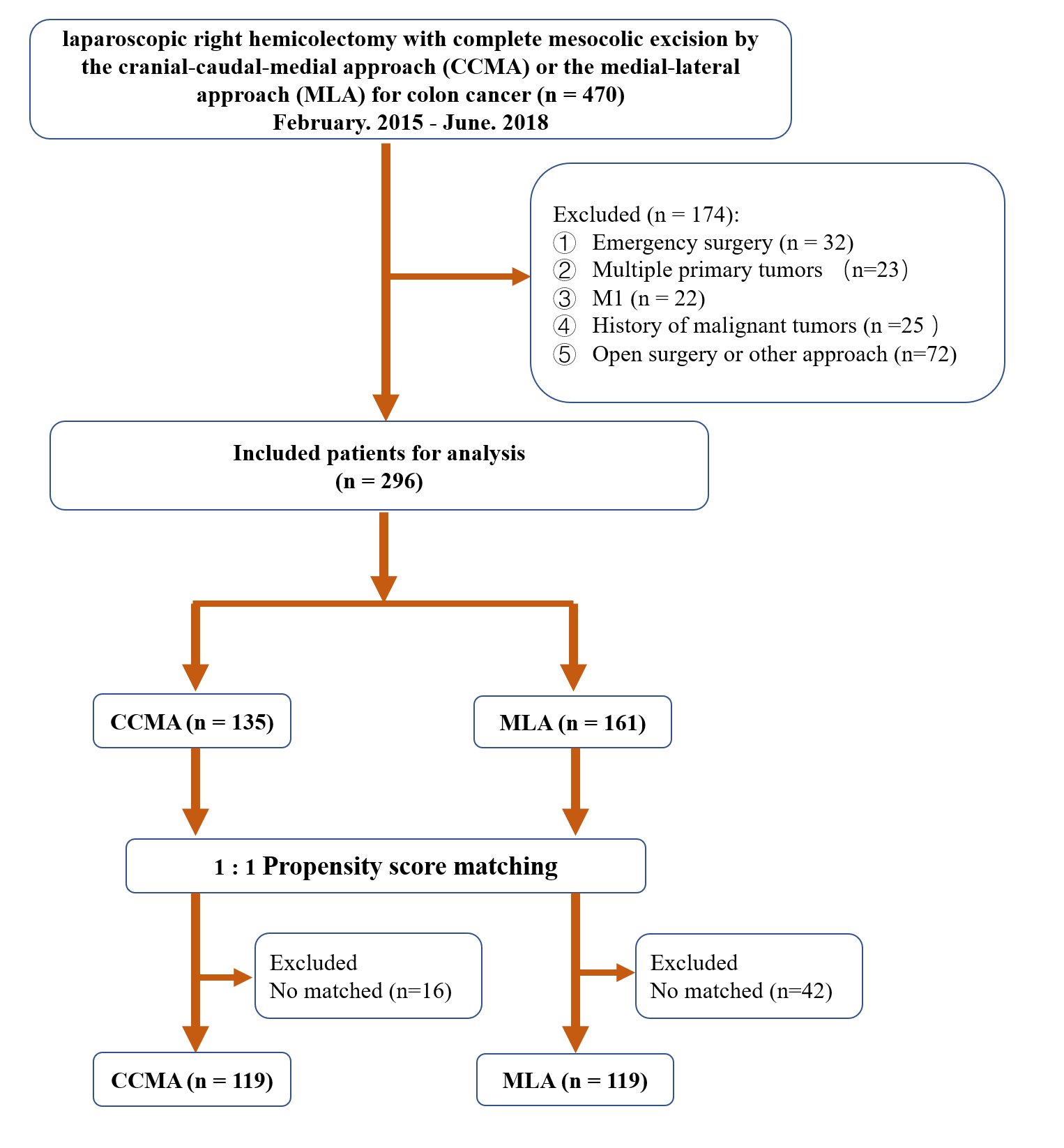


**Supplementary Table 1.** **Cox regression analysis of the OS and DFS (Supplementary data).**

| **Characteristics** | **OS** | | | | **DFS** | | | |
| --- | --- | --- | --- | --- | --- | --- | --- | --- |
|  | **Univariate analysis** | | **Multivariate analysis** | | **Univariate analysis** | | **Multivariate analysis** | |
|  | **HR (95%CI)** | ***P*** | **HR (95%CI)** | ***P*** | **HR (95%CI)** | ***P*** | **HR (95%CI)** | ***P*** |
| **Sex** |  |  |  |  |  |  |  |  |
| **Female** | Reference | - | - | - | Reference | - | - | - |
| **Male** | 0.805 (0.461-1.406) | 0.445 | - | - | 0.529 (0.266-1.053) | 0.070 | - | - |
| **Age** | 1.062 (1.020-1.105) | 0.003** | 1.043 (1.005-1.083) | 0.028* | 1.049 (1.003-1.097) | 0.037* | 1.032 (0.990-1.077) | 0.137 |
| **BMI** |  |  |  |  |  |  |  |  |
| **＜28.0 kg/m2** | Reference | - | - | - | Reference | - | - | - |
| **≥28.0 kg/m2** | 1.254 (0.391-4.025) | 0.703 | - | - | 1.142 (0.415-3.146) | 0.797 | - | - |
| **ASA classification** |  |  |  |  |  |  |  |  |
| **Ⅰ** | Reference | - | - | - | Reference | - | - | - |
| **Ⅱ** | 0.955 (0.530-1.719) | 0.877 | - | - | 1.184 (0.570-2.459) | 0.650 | - | - |
| **Ⅲ** | 0.700 (0.281-1.743) | 0.443 | - | - | 1.485 (0.585-3.772) | 0.406 | - | - |
| **Tumor differentiation** |  |  |  |  |  |  |  |  |
| **High** | Reference | - | - | - | Reference | - | - | - |
| **Moderately** | 1.498 (0.448-5.005) | 0.512 | 1.396 (0.413-4.717) | 0.591 | 1.429 (0.325-6.290) | 0.637 | 1.319 (0.297-5.858) | 0.716 |
| **Poorly** | 5.384 (1.601-6.101) | 0.007** | 2.984 (0.856-7.404) | 0.086 | 6.095 (1.390-5.735) | 0.017* | 3.851 (0.853-7.386) | 0.080 |
| **Mucous** | 0.381 (0.091-1.594) | 0.186 | 0.472 (0.107-2.078) | 0.321 | 0.710 (0.143-3.520) | 0.675 | 1.074 (0.202-5.721) | 0.933 |
| **No. of lymph nodes harvested** | 0.983 (0.941-1.027) | 0.436 | - | - | 0.980 (0.930-1.033) | 0.450 | - | - |
| **Perineural invasion** |  |  |  |  |  |  |  |  |
| **No** | Reference | - | - | - | Reference | - | - | - |
| **Yes** | 2.491 (1.388-4.473) | 0.002** | 2.140 (1.119-4.091) | 0.021* | 2.322 (1.164-4.633) | 0.017* | 1.863 (0.896-3.874) | 0.096 |
| **Vascular invasion** |  |  |  |  |  |  |  |  |
| **No** | Reference | - | - | - | Reference | - | - | - |
| **Yes** | 2.559 (1.400-4.679) | 0.002** | 2.948 (1.522-5.711) | 0.001** | 2.252 (1.089-4.657) | 0.029* | 2.301 (1.062-4.984) | 0.035* |
| **Pathological TNM** |  |  |  |  |  |  |  |  |
| **II** | Reference | - | - | - | Reference | - | - | - |
| **III** | 2.919 (1.552-5.489) | 0.001** | 2.298 (1.155-4.572) | 0.018* | 3.899 (1.702-8.931) | 0.001 | 3.425 (1.398-8.390) | 0.007** |
| **Approach** |  |  |  |  |  |  |  |  |
| **MLA** | Reference | - | - | - | Reference | - | - | - |
| **CCMA** | 1.170 (0.678-2.018) | 0.573 | - | - | 1.148 (0.697-1.891) | 0.588 | - | - |

Results are expressed as HR (95%CI). **P*<0.05, ***P*<0.01, ****P*<0.001.

Abbreviations: OS overall survival, DFS disease-free survival, HR hazard ratio; CI confidence interval, ASA American Society of Anesthesiologists, CCMA cranial-caudal-medial approach, MLA medial-lateral approach.

**Supplementary Table 2. Recurrence patterns (Supplementary data).**

| **Characteristics** | **CCMA group**  **(n=119)** | **MLA group**  **(n=119)** |
| --- | --- | --- |
| **Recurrence (n, %)** |  |  |
| **Yes** | 33 (27.7%) | 29 (24.4%) |
| **No** | 86 (72.3%) | 90 (75.6%) |
| **Locoregional only (n, %)** | 10 (7.56%) | 8 (5.88%) |
| **Distant only (n, %)** | 13 (10.92%) | 11 (9.24%) |
| **Locoregional +distant (n, %)** | 10 (8.40%) | 10 (8.40%) |
| **Sites of distant (n, %)** |  |  |
| **Liver (n, %)** | 10 (8.4%) | 9 (7.6%) |
| **Peritoneal (n, %)** | 6 (5.0%) | 5 (4.2%) |
| **Lung (n, %)** | 10 (8.4%) | 8 (6.7%) |
| **Retroperitoneum (n, %)** | 2 (1.7%) | 3 (2.5%) |
| **Brain (n, %)** | 1 (0.8%) | 0 |
| **Ovary (n, %)** | 1 (0.8%) | 1 (0.8%) |

Results are expressed as n (%).

Abbreviations: CCMA cranial-caudal-medial approach, MLA medial-lateral approach.
